# 

**Published:** 1905-07

**Authors:** 


### CONTENTS.

ORIGINAL COMMUNICATIONS—

Cystitis. By E. G. Ballenger, M.D., Atlanta, Ga. .......... 217

Personal Views of the Management of Typhoid Fever. By Edward C. Register, M.D., Charlotte, N. C. .......... 226

EDITORIAL NOTES AND COMMENTS—

The Origin of Syphilis .......... 243

Report of Georgia State Commission on Tuberculosis. By Chas. Hicks, M.D., Chairman, Dublin, Ga. 245

Transactions of the Georgia State Commission on Tuberculosis .......... 251

Secretary's Report Georgia State Commission on Tuberculosis .......... 257

NEWS AND NOTES— .......... 265

CONTENTS CONTINUED .......... X

